# Thirty-year clinical experience in gamma knife radiosurgery for trigeminal schwannomas

**DOI:** 10.1038/s41598-022-18689-5

**Published:** 2022-08-23

**Authors:** Dong-Won Shin, Chunseng Ju, Hyun Seok Lee, Hee Jun Yoo, Sang Woo Song, Young Hyun Cho, Chang-Ki Hong, Seok Ho Hong, Do Heui Lee, Jeong Hoon Kim, Young-Hoon Kim

**Affiliations:** 1 Department of Neurological Surgery, Asan Medical Center, University of Ulsan College of Medicine, 88, Olympic-ro 43-gil, Songpa-gu, Seoul, 05505 Republic of Korea; 2 Department of Neurosurgery, Gil Medical Center, Gachon University College of Medicine, Incheon, Republic of Korea; 3 Department of Plastic Surgery, Suwon Ever Plastic Surgery Clinic, Suwon, Gyeonggi-do Republic of Korea; 4 Department of Neurosurgery, Konkuk University Hospital, Seoul, Republic of Korea; 5 Department of Neurosurgery, Chung-Ang University Gwangmyeong Hospital, Gwangmyeong, Gyeonggi-do Republic of Korea

**Keywords:** Cancer, Neurology, Oncology

## Abstract

We aimed to evaluate the radiographic and clinical outcomes after gamma knife radiosurgery (GKRS) for trigeminal schwannomas (TSs). A total of 87 patients who underwent GKRS for TSs between 1990 and 2020 were enrolled. The mean tumor volume was 4.3 cm^3^. The median prescribed dose for the margins of the tumor was 13 Gy. The median follow-up duration was 64.3 months (range 12.0–311.5 months). The overall local tumor control rate was 90%, and the symptom response rate was 93%. The response rate for each symptom was 88% for facial pain, 97% for facial sensory change, and 86% for cranial nerve deficits. Nineteen (22%) patients showed transient swelling, which had regressed at the time of the last follow-up. Cystic tumors were associated with transient swelling (*p* = 0.04). A tumor volume of < 2.7 cm^3^ was associated with local tumor control in univariable analysis. Transient swelling was associated with symptom control failure in both univariable and multivariable analyses (*p* = 0.04, odds ratio 14.538). GKRS is an effective treatment for TSs, both for local control and symptom control.

## Introduction

Trigeminal schwannomas (TSs) are rare intracranial neoplasms, accounting for < 0.5% of all intracranial tumors^1–4^. Eisenberg et al. surgically removed 40 tumors of the cavernous sinus and reported that 13 tumors were TSs^2^. TSs usually occur in Meckel’s cave or in the prepontine cisternal space; however, they often invade the cavernous sinus and extracranial space. Owing to their rarity, TSs have rarely been the subject of clinical studies, compared with vestibular schwannomas (VSs). Although advanced techniques have reduced the surgical morbidity of skull base surgery, tumors located in the cavernous sinus or Meckel’s cave still present a surgical challenge. Despite the increasing popularity of endoscopic endonasal and transorbital approaches, problems including insufficient experience and lack of studies with a long-term follow-up remain^5–8^. As gamma knife radiosurgery (GKRS) has demonstrated successful tumor control in VSs, expanding its application to TSs appears to be a natural development. Therefore, we performed this large single-institution study with a long-term follow-up to evaluate the radiographic and clinical outcomes after GKRS for TSs.

## Methods

All procedures performed in studies involving human participants were in accordance with the ethical standards of the institutional review board of the Asan Medical Center and with the 1964 Helsinki declaration and its later amendments or comparable ethical standards. The experimental protocol was approved by the Institutional Review Board of the Asan Medical Center (IRB number: 2021-1767). Because of its retrospective manner, informed consent was waived.

### Patient population

Between September 1991 and June 2020, a total of 100 patients with TSs underwent GKRS at our institution. Of them, we excluded four patients for having a follow-up of < 12 months, one for having a postoperative pathologic diagnosis other than TS, five for being duplicate cases, and three for having an ambiguous radiographic diagnosis. After excluding these 13 patients, 87 (88 tumors) were finally included in this study.

### Radiosurgical technique

Radiosurgery was performed using a Leksell Gamma Knife (Models B, C, and Perfexion; Elekta, Stockholm, Sweden). Leksell Gamma knife type B was utilized from 1991 to 2003, type C was utilized from 2003 to 2011, and Perfexion was utilized from 2011 to 2020. The procedure started with placement of a Leksell stereotactic frame while the patient was under local scalp anesthesia. Thereafter, a gadolinium contrast magnetic resonance T1-weighted image with 2-mm slice reconstruction on the axial plane was obtained. The image was exported to a computer workstation via the hospital Ethernet for use in dose planning. Leksell GammaPlan software (version 10.2.1, Elekta) was used to accurately calculate the amount of radiation received at each anatomical point and to subsequently generate a distance–dose curve for the radiation dosage. The mean tumor volume was 4.3 cm^3^ (0.08–19.9 cm^3^). The median prescription dose delivered to the margins of the tumor was 13 Gy (10–18 Gy) for a single fraction. The prescription isodose was 50% in 81 (93%) patients. The maximum radiation dose varied from 20 to 42 Gy (median 26 Gy). Three (3%) patients underwent fractionated GKRS. Fractionated GKRS was performed in selective cases who had large tumor volume. Among three patients, two underwent 3 fractions (21 Gy/3fx, 50% isodose line), and the other one underwent 5 fractions (26.5 Gy/3fx, 50% isodose line). Seventy patients (87%) underwent GKRS as an initial treatment, whereas 11 (13%) had undergone surgical resection before GKRS. The patients underwent GKRS either as an outpatient procedure or during admission with discharge at 24–48 h after the procedure.

### Follow-up review

After GKRS, the patients were instructed to undergo clinical and imaging assessments at 6-month intervals during the first year and annually thereafter for 2 years. If tumor growth was halted, additional imaging evaluations were scheduled at 4, 6, 8, and 12 years after GKRS. If a new neurologic symptom or sign developed, the patient was evaluated for tumor progression or any adverse effects of radiation, and a new magnetic resonance image was obtained. Follow-up magnetic resonance images were compared with the intraoperative images, and tumor dimensions were measured in the axial, sagittal, and coronal planes. All images were reviewed by neurosurgeons and neuroradiologists, and computer-based tumor volumetric measurements were performed. The tumor size measured on the images was classified as increased, stable, or decreased. The images were also assessed for central necrosis or transient swelling. To assess clinical symptoms, the electronic medical records were carefully reviewed, and the clinical course of the initial symptoms was observed. The symptoms were classified as improved, stable, or worsened. If the patients needed a higher dose of medication (e.g., gabapentin, carbamazepine, or pregabalin) than before radiosurgery, they were considered to have worsened symptoms.

### Statistical analyses

Patient- or tumor-related factors were included in the statistical analyses. Correlation analysis was performed for clinical factors. Pearson’s correlation coefficients were used to assess the relationship among factors. Binary logistic regression analysis was performed for univariable and multivariable analyses. Logistic regression analysis was performed for each clinical factors followed by multivariable analysis according to the result of univariate analysis. Kaplan–Meier survival analysis was performed for tumor progression free period. Data analysis was performed using IBM SPSS Statistics (version 23; IBM Corp.) and R studio (version 22.02.1). All tests were two-sided, and *p* < 0.05 was considered to indicate statistical significance.

## Results

### Patients’ characteristics

**Figure 1:**
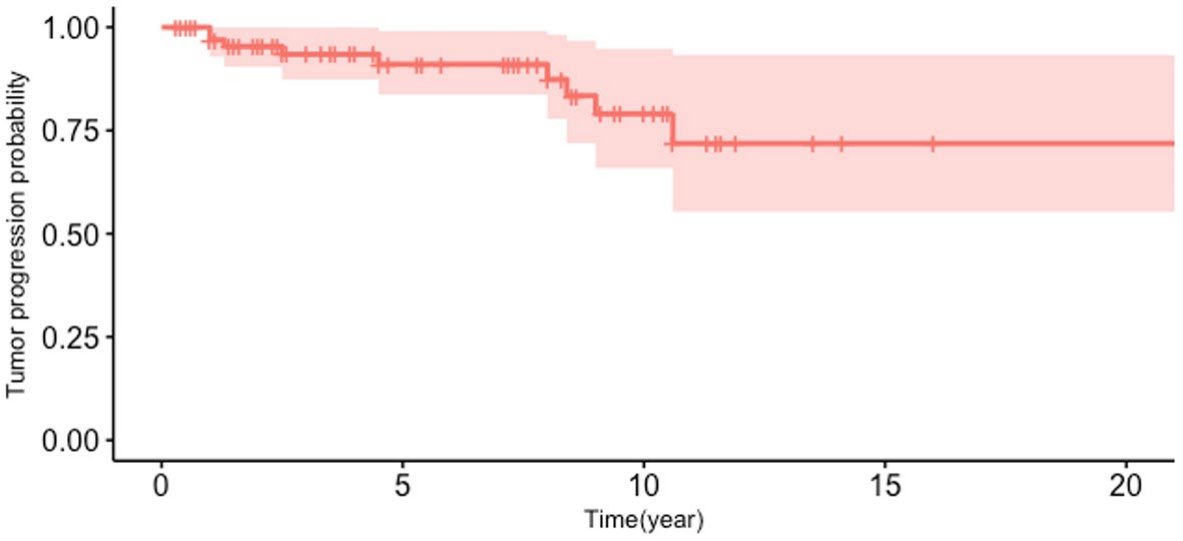
Kaplan–Meier survival curve analysis after GKRS for trigeminal schwannomas.

The median age of the patients was 53 years (range 13–79 years), and the median follow-up period was 64.3 months (range 12.0–311.5 months). The male-to-female ratio was 37:50. One patient had neurofibromatosis type 2. In addition, 17 (20%) patients had hypertension and 5 (6%) had diabetes mellitus. Thirteen (15%) patients were prescribed medications for symptom control. Table 1 shows the demographic characteristics of our study patients. Kaplan–Meier curve for the tumor progression-free rate was shown in Fig. 1.

**Table 1:** Demographic characteristics of patients.

No. of patients	87 (88 tumors)
Median follow-up period	64.3 months (12.0–311.5 months)
Median age	53 years (13–79 years)
Male:female	37:50
Neurofibromatosis type 2	1
Operation history	11 (13%)
Hypertension	17 (20%)
Diabetes mellitus	5 (6%)
Medication use	13 (15%)
**Radiosurgery details**	
Mean tumor volume	4377 mm^3^
Dose	13 Gy (50% isodose line)
Fractionation	3
Repeat GKRS	4
**Tumor type**	
Root	37 (43%)
Ganglion	32 (36%)
Dumbbell	19 (22%)
Tumor location	
Cisternal	32 (36%)
Meckel’s cave	41 (47%)
Cavernous sinus	5 (6%)
Foraminal	9 (10%)
Extracranial	1 (1%)
**Imaging findings**	
T1 signal intensity	
Low	41
Iso	32
High	0
T2 signal intensity	
Low	2
Iso	44
High	29
Extracranial extension	10 (11%)
Cystic portion	13 (15%)
Transient swelling	19 (22%)
Central necrosis	37 (42%)
Peritumoral edema	1 (1%)
**Outcomes**	
Imaging	
Shrunk	40 (45%)
Stable	39 (44%)
Progressed	9 (10%)
Symptom	
Improved	44 (50%)
Stable	38 (43%)
Aggravated	6 (7%)

### Imaging findings

Thirty-seven (42%) patients had a root-type tumor, whereas 32 (36%) had a ganglion-type tumor. The most common location was Meckel’s cave (46%), followed by the cisternal space (36%). Ten (11%) patients had an extracranial extension, and 13 (17%) had a cystic portion inside the tumor. During the follow-up period, transient swelling was observed in 19 (22%) patients, whereas central necrosis (loss of mural enhancement) was noted in 37 (42%). Transient swelling developed at a median of 4.9 months after GKRS (range 2.6–10.4 months) and resolved at a median of 20.1 months after GKRS (range 6.0–37.0 months). The median duration of transient swelling was 15.2 months (range 2.9–34.4 months). Central necrosis developed at a median of 6 months after GKRS (range 2.5–18.0 months). Peritumoral edema was observed in only one patient. Most tumors showed low to iso signal intensity on T1-weighted images and iso to high signal intensity on T2-weighted images.

Of the tumors, 40 (45%) showed shrinkage, 39 (44%) remained stable, and 9 (10%) progressed after GKRS. Among 9 patients who had tumor progression, 5 (56%) patients showed tumor expansion in 2 years after GKRS, one patient in 5 years, and three patients in 8.7, 10.6, and 12.7 years. Of the nine patients who showed disease progression after GKRS, only two underwent GKRS again and two other patients underwent surgical removal. The overall radiological and clinical tumor control rates were 90 and 95%, respectively. However, the 1,5,10, and 15-year tumor progression-free rates were 96.2%, 91.2%, 87.4%, and 73.7%, respectively in the Kaplan–Meier curve (Fig. 1).

**Figure 2:**
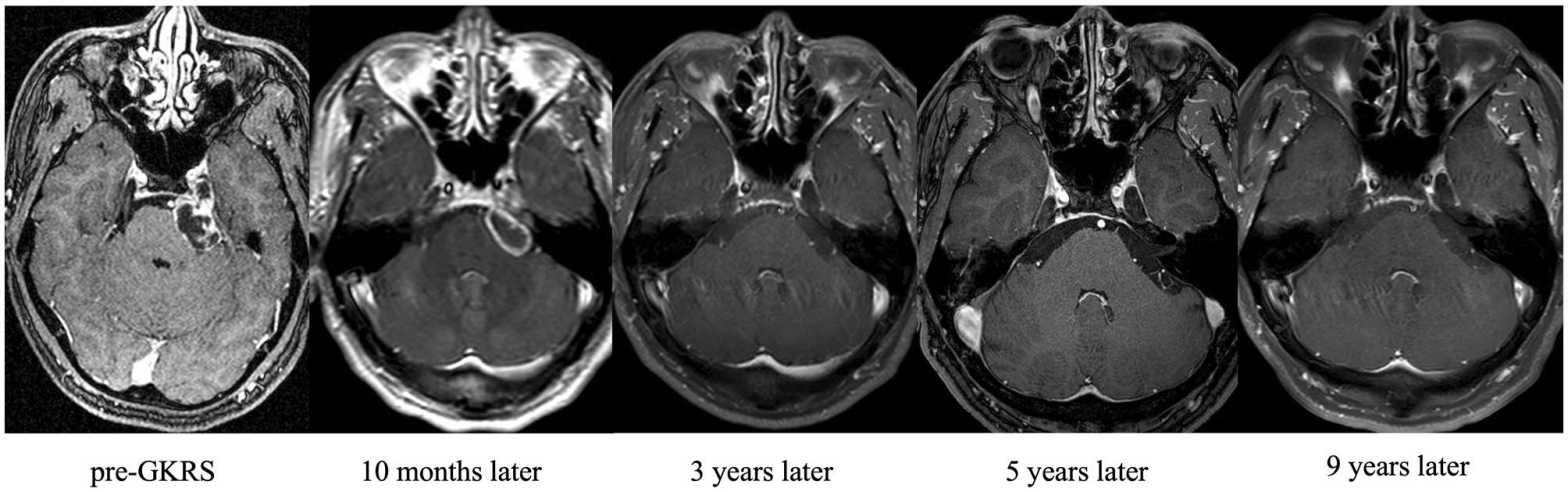
Illustrative case showing a gradual decrease in tumor size after GKRS (12 Gy, 50% isodose line, 6.3 cm^3^). A 43-year-old male improved facial hypesthesia as well as radiographic finding 9 years after GKRS. Initial MRI showed the tumor occupied Meckel’s cave and prepontine cistern, and compressed brainstem. It was gradually decreased, and it was completely diminished at 9 years post-GKRS. GKRS, gamma knife radiosurgery.

Figure 2 showed a dumbbell-shape TS that was well-controlled in both the clinical symptom and radiographic outcome at 9 years after GKRS. Local control was associated with tumor volume < 2.7 cm^3^ in univariable analysis, although statistical significance was reached only in multivariable analysis. Table 2 shows the results of univariable and multivariable analyses.

**Table 2:** Results of univariable and multivariable analyses of symptom control and local control.

Factors	Univariable analysis			Multivariable analysis		
	*p* value	OR	95% CI	*p* value	OR	95% CI
**Symptom control**						
**Sex**						
Male	–	–	–			
Female	0.22	4.00	0.45–35.79			
**Age**						
< 50	–	–	–	–		
≥ 50	0.05	0.11	0.01–1.00	0.99		
Operation	0.99					
Hypertension	0.99					
Diabetes mellitus	0.99					
**Volume**						
< 2.7 cm^3^	–	–	–			
≥ 2.7 cm^3^	0.44	2.00	0.35–11.53			
**Fraction**						
No	**–**	–	–	–		
Yes	0.01*	40.50	3.00–546.33	0.99		
Tumor type	0.67	0.79	0.26–2.41			
Tumor location	0.15	1.71	0.83–3.54			
Extracranial extension	0.67	1.62	0.17–15.48			
T1 signal intensity	0.19	0.23	0.03–2.10			
T2 signal intensity	0.90	0.90	0.19–4.33			
Cystic portion	0.29	2.68	0.44–16.48			
**Transient swelling**						
No	**–**	–	–	**–**	–	–
Yes	**0.01**	10.18	1.66–62.61	**0.04**	14.54	1.06–200.27
Central necrosis	0.13	5.47	0.61–49.35			
**Local control**						
Controlled	–	–	–	–	–	–
Failed	0.08	5.36	0.83–34.61	0.09	11.41	0.67–194.42
Medication	0.99					
**Local control**						
Sex	0.90	0.92	0.23–3.68			
**Age**						
< 50	–	–	–	–	–	–
≥ 50	0.09	0.28	0.07–1.21	0.23	0.38	0.08–1.81
Operation	0.37	2.19	0.39–12.21			
Hypertension	0.51	0.48	0.06–4.16			
Diabetes mellitus	0.99					
**Volume**						
< 2.7 cm^3^	**–**	**–**	**–**	**–**	**–**	**–**
≥ 2.7 cm^3^	**0.04**	9.08	1.08–76.06	0.05	8.47	0.98–73.17
Fraction	0.99					
Tumor type	0.41	1.45	0.60–3.50			
Tumor location	0.61	0.82	0.37–1.80			
Extracranial extension	0.98	0.97	0.11–8.70			
T1 signal intensity	0.71	1.32	0.30–5.75			
T2 signal intensity	0.61	1.40	0.38–5.22			
Cystic portion	0.99					
Transient swelling	0.56	0.52	0.06–4.59			
Central necrosis	0.14	0.29	0.05–1.52			
**Symptom control**						
Yes	–	–	–	–	–	–
No	0.08	5.36	0.83–34.61	0.24	3.42	0.44–26.39
Medication	0.80	0.75	0.08–6.82			

*Bold *p* values are statistically significant.

### Clinical factor analysis

We performed correlation analysis and assessed Pearson’s correlation coefficients among the clinical factors. Patients with diabetes mellitus needed medications for pain control (*p* = 0.01). Transient swelling was associated with a cystic tumor (*p* = 0.04).

### Symptom outcome

**Figure 3:**
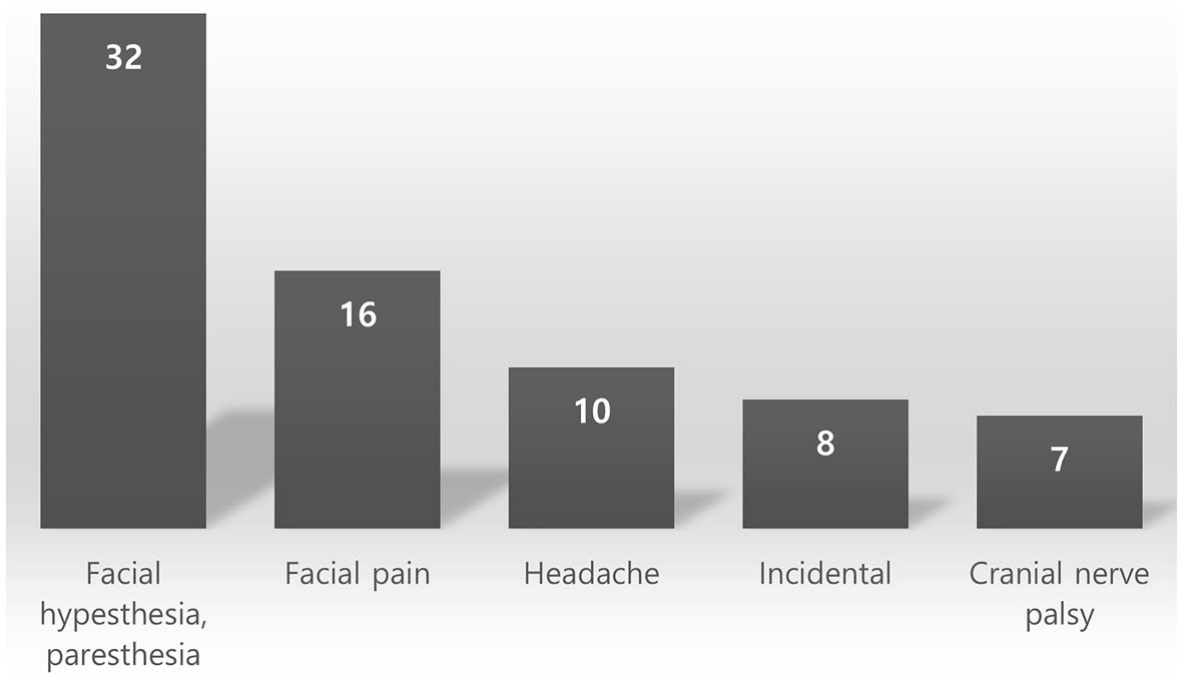
Symptom distribution in patients with trigeminal schwannomas.

Facial pain and sensory change (66%) were the most common symptoms, followed by headache (14%). Seven (10%) patients presented cranial nerve deficits. Eight (11%) patients had no symptoms before GKRS. Figure 3 describes the symptom distribution in patients with TSs. No patient needed an increase in the medication dose during the follow-up period. The symptom outcomes after GKRS are summarized in Table 3. The symptoms improved in 18 (33%) patients, remained stable in 33 (60%), and worsened in 4 (7%). In univariable analysis, fractionated GKRS and transient swelling were associated with symptom control failure. Additionally, elderly patients (age more than 50) showed better symptom control than younger patients (*p* = 0.05). In multivariable analysis, transient swelling was associated with symptom control failure (*p* = 0.045, odds ratio 14.538, 95% confidence interval 1.055–200.268). The clinical course of each symptom after GKRS is described below.

**Table 3:** Symptom outcome after gamma knife radiosurgery for trigeminal schwannomas.

Symptoms	Status			Total
	Improved	Stable	Worsened	
Facial pain	6 (38%)	8 (50%)	2 (13%)	16
Facial hypesthesia/paresthesia	10 (31%)	21 (66%)	1 (3%)	32
Cranial nerve palsy	2 (29%)	4 (57%)	1 (14%)	7
Total	18 (33%)	33 (60%)	4 (7%)	55

### Facial sensory change (paresthesia or hypesthesia)

Thirty-two (44%) patients presented either paresthesia or hypesthesia. The symptom duration was from 1 week to 84 months. Six patients needed medications: four were prescribed gabapentin, one was prescribed topiramate, and one was prescribed both oxcarbazepine and gabapentin. Symptom relief after GKRS was observed starting from 1 to 28 months. Of the 32 patients, 31 had improved or stable symptoms after GKRS; however, one patient experienced facial paresthesia worsening. A 54-year-old female patient who developed mastication problem, diplopia, and facial hypesthesia at 8 years after GKRS. In this patient, symptom progression was accompanied by tumor progression or hemorrhagic transformation. Of the eight patients who did not present any symptom before GKRS, one experienced facial sensory change after treatment.

### Facial pain

**Figure 4:**
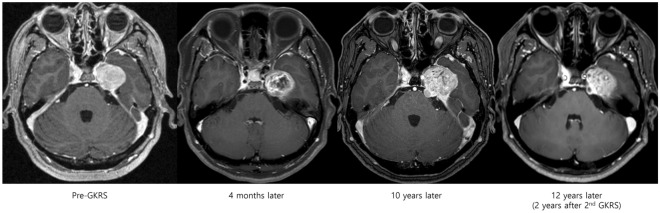
Illustrative case showing tumor size reduction and symptom improvement at 4 months after GKRS (12 Gy, 50% isodose line). The patient experienced facial pain worsening at 10 years after GKRS. Brain magnetic resonance imaging shows tumor progression. The patient underwent a second GKRS (13 Gy, 50% isodose line) and was followed up. *GKRS* gamma knife radiosurgery.

Sixteen (22%) patients presented facial pain. The symptom duration was from 3 to 60 months. Six patients needed medications: two were prescribed carbamazepine, two were prescribed gabapentin, one was prescribed pregabalin, and one was prescribed both carbamazepine and gabapentin. Of the 16 patients with facial pain, 6 (38%) showed symptom improvement, 8 (50%) had a stable symptom, and 2 (13%) had a worsened symptom. Facial pain improvement mostly occurred within 16 months except in one patient who experienced pain improvement immediately after GKRS. Among patients with a worsened symptom, one developed diplopia and facial pain worsening 2.5 years after GKRS. Follow-up magnetic resonance imaging revealed central necrosis and volume expansion. Figure 4 shows the images from a 49-year-old female patient who experienced symptom aggravation due to tumor progression at 10 years after GKRS. Pain aggravation was also accompanied by tumor volume expansion in both patients with worsened facial pain. Among the 71 patients who did not have facial pain before GKRS, 17 (24%) developed transient pain and 2 (3%) experienced progressively worsening pain after GKRS.

### Cranial nerve palsy

Seven patients had cranial nerve deficits before GKRS. Four patients had diplopia, two had facial palsy, and one had abducens nerve palsy. One patient fully recovered from diplopia 1 year after GKRS, and two had a stable symptom. In one patient, diplopia immediately improved after GKRS; however, tumor necrosis led to worsening of diplopia at 4 months after GKRS, which was treated with ophthalmologic surgical repair. Of the two patients who had facial palsy, one experienced facial palsy aggravation 2 days after GKRS, which improved in 2 months, whereas the other patient had a stable symptom. Abducens nerve palsy immediately improved after GKRS, and the patient fully recovered in 22 months.

## Discussion

Surgical resection of TSs can result in postoperative neurologic deficits in 13%–86% of the cases^3,9,10^. Al-Mefty et al. reported that trigeminal symptoms were improved in 44%, facial pain was ameliorated in 73%, and trigeminal motor symptoms were improved in 80% of their patients after surgical resection. Surgery-induced cranial nerve dysfunction occurred in a total of 11 patients (44%)^9^. Owing to the high rate of surgical morbidity and difficulty in the surgical approach, TSs have presented a challenge to neurosurgeons. Recently, advanced techniques, such as endoscopic skull base surgery, have encouraged neurosurgeons to approach the infratemporal fossa or Meckel’s cave^5–7^. The endoscopic approach has major advantages of minimal invasiveness, avoidance of a large craniotomy, fewer cosmetic problems, and tumor removal along a parallel axis. In a retrospective multicenter study that enrolled 25 patients, gross total resection or near-total resection was achieved in 19 (76%) with complications in three (12%) patients^6^.

### Stereotactic radiosurgery

The successful local control of VSs using GKRS, which is a form of stereotactic radiosurgery, has encouraged extending the application of GKRS to the treatment of TSs. The local control rate of TSs was 77.3–88% in many studies^3,4,11,12^, which was comparable to the control rate of other benign tumors, such as meningiomas or VSs. Kano et al. reported an 87.9% tumor control rate after stereotactic radiosurgery. They also found that target volume, male sex, and dumbbell-type tumors were associated with worse progression-free survival^3^. In this study, sex or tumor type were associated with neither symptom control nor local control. Among nine patients who were radiographically progressed after GKRS, only four patients needed further management so that clinical tumor control rate was up to 95%. However, as we can find in Kaplan–Meier curve for the tumor progression-free period, long term tumor control rate (over 15 years) was less than 75%. We emphasized that long-term follow-up was mandatory if possible after GKRS though tumor volume was decreased. Physicians should educate the patients not to lose their follow up.

Transient swelling was observed in 19 (22%) patients; however, the follow-up period may be a major factor in differentiating between transient swelling and tumor progression; that is, if increased tumor volume was observed at a certain follow-up time point or in the last follow-up, it was defined as tumor progression. However, if tumor shrinkage was observed after a certain time point, it was defined as transient swelling. As tumor progression and transient swelling may follow the same volume expansion after GKRS, the timing of data collection is an important factor in differentiating between these two outcomes. We thought that the initial 2 to 5 years after GKRS is a critical period for both symptomatic and image outcomes. During that period, the physician should educate the patients that they can develop both symptomatic progression and radiographical aggravation.

### Transient swelling

Transient tumor expansion or transient swelling is a key concern for clinicians. Physicians sometimes faced unexpected or sudden volume expansion of tumors during the follow-up period after GKRS. It is difficult to determine whether it is true progression or not. Moreover, if the patients had symptom aggravation, the situation went worse. Although several possible mechanisms have been proposed, the exact mechanism remains unclear^13–15^. Nagano et al. have shown that peak expansion was observed at 6.4 months after GKRS. Among the tumors, half returned to their initial size within 1 year. The authors also found that high-dose treatment was significantly related to tumor expansion, although without statistical significance. Moreover, clinical aggravation was correlated with tumor expansion^16^. In our study, transient swelling was observed in 19 (22%) patients. The median time to the development of transient swelling was 4.9 months, and the tumors returned to their initial size within 20.1 months after GKRS. Cystic tumors were associated with transient swelling in correlation analysis (*p* = 0.04). In multivariable analysis, transient swelling was related to poor symptom control (*p* = 0.04, odds ratio 14.538). We need to educate the patients about the worsening symptoms due to transient swelling which usually occurs between 5 and 20 months after GKRS. In this period, we prescribed dexamethasone, carbamazepine, or other drugs for neuropathic pain.

### Symptom control after GKRS

Kano et al. found that patients without prior surgical resection had significantly better improvement in neurologic symptoms and signs (*p* = 0.04)^3^. Ryu et al. reported their clinical outcome of 32 patients with TSs. They found 12 of 26 patients with trigeminal sensory disturbance, 9 of 11 with trigeminal pain, and two of seven with ocular symptoms showed symptom improvement^17^. As in our study, ocular symptoms, such as diplopia or cranial nerve palsies (involving the third, fourth, or sixth nerves), were among the major concerns with the application of GKRS in their study. In present study, diplopia was observed in four patients. One patient had symptom aggravation due to tumor necrosis, which was treated with surgical correction in the department of ophthalmology. Two patients had stable symptoms after GKRS. One patient disappeared their symptom in 1 year after GKRS. The clinical response of cranial nerve palsy varied among patients or depending on the damaged nerve. It was difficult to predict the clinical outcome of cranial nerve palsy after GKRS.

Snyder et al. achieved symptom improvement in 42.1% and symptom stability in 42.1% of their patients. Facial pain was improved in 4 of 7 (57.1%) patients, whereas facial numbness was improved in 5 of 14 (35.7%)^4^. In this study, facial pain and facial numbness were improved in 37.5% and 31.3% of the patients, respectively. Snyder et al. also reported that three (15.8%) patients had symptom progression at a median of 4 months (range 1–40 months). In this study, symptom improvement was achieved in 18 of 55 (32.7%) patients, whereas the symptoms were stable in 33 (60%). During the follow-up period, none of the patients needed an increased medication dose. In one patient, the medication was changed from carbamazepine to gabapentin because of the drug's adverse effects. In detail, facial sensory change (response rate 96%) showed the best treatment response, followed by facial pain (response rate 88%).

Unfortunately, three of eight (37.5%) patients who were incidentally found to have TSs developed new symptoms after GKRS. Clinicians should carefully inform the patients about possible clinical outcomes, such as facial sensory change, transient cranial nerve palsy, or diplopia before they undergo GKRS. If the symptoms worsen at any time after GKRS, mandatory imaging follow-up may be needed. Internal hemorrhage or tumor progression can develop at 10 years after GKRS or even when the imaging examination in the previous year showed stable symptoms. We found that clinical deterioration was usually associated with tumor progression, necrosis, or hemorrhage, which can occur at any time after GKRS.

### Limitations

The diagnosis of TSs was mostly based on radiographic findings. The differential diagnosis between transient swelling and tumor progression was challenging, and the follow-up period may be a key contributing factor.

Moreover, symptom control was subjective and differed depending on the patients’ characteristics, whereas local control was more objectively assessed using radiographic findings. A pain scale can provide more objective results than subjective descriptions by the patients themselves. This study's definition of symptom response was an improvement or a stable status of symptoms and a reduction in the medication dose. This definition may be inappropriate or can lead to misinterpretation. Whether to include improved symptoms alone or both improved and stable symptoms in the definition of symptom control is ambiguous.

## Conclusions

GKRS is an effective and non-invasive treatment method for TSs. Transient swelling was associated with symptom control failure, whereas tumor volume ≥ 2.7 cm^3^ was associated with local control failure in our study. Patients with cystic tumors tended to develop transient swelling after GKRS. Therefore, the risk–benefit ratio of GKRS for cystic TSs should be considered. If tumor volume increased in 5 years of GKRS, determination between transient swelling and tumor progression was difficult so frequent follow-up and symptomatic control were recommended at that period. In appropriately selected patients, GKRS is an excellent treatment modality for both symptom control and local control.

## Data Availability

All data included in this study can be provided by contacting dwshinns@gmail.com.
